# Tenecteplase or Alteplase: What Is the Thrombolytic Agent of the Future?

**DOI:** 10.1007/s11940-022-00733-4

**Published:** 2022-07-30

**Authors:** Ammad Mahmood, Keith W. Muir

**Affiliations:** 1 School of Psychology and Neuroscience, Queen Elizabeth University Hospital, University of Glasgow, Glasgow, UK; 2 School of Psychology and Neuroscience, Queen Elizabeth University Hospital, University of Glasgow, 1345 Govan Road, Glasgow, G51 4TF UK

**Keywords:** Tenecteplase, Alteplase, Acute ischaemic stroke, Thrombolysis

## Abstract

**Purpose of review:**

Alteplase has been the thrombolytic of choice for acute ischaemic stroke for more than two decades. A thrombolytic which is easier to administer and with improved or comparable safety and efficacy is desirable. Tenecteplase has emerged as a potential successor, and its off-license use in acute ischaemic stroke has increased in recent years. We aimed to examine the evidence base for each drug and discuss their use in varying patient populations in acute ischaemic stroke.

**Recent findings:**

Several trials comparing tenecteplase and alteplase have reported very recently with the results of the ACT trial strengthening the argument in favour of non-inferiority of tenecteplase to alteplase. Ongoing trials such as ATTEST-2 are of interest, and trials such as TASTE and TEMPO-2 will shed further light on use of tenecteplase in specific populations.

**Summary:**

A single thrombolytic agent for all indications for thrombolysis in acute ischaemic stroke is desirable in streamlining workflows. Based on recent and upcoming trials, guidelines may soon recommend tenecteplase as a suitable alternative to alteplase. The use of tenecteplase in specific subgroups will depend on further recruitment to ongoing clinical trials.

## Introduction

Stroke is the second-leading cause of death and third-leading cause of death and disability globally, with 12.2 million incident cases of stroke and 6.55 million deaths from stroke in 2019 [1]. Ischaemic stroke is the commonest type of stroke globally (62.4%). In the first hours after onset, management of potentially disabling acute ischaemic stroke (AIS) focuses on early reperfusion of ischaemic brain. Reperfusion can limit the extent of brain tissue death by rescuing ischaemic tissue with residual perfusion that is sustained by collateral flow, which typically remains viable for a period of a few hours only. Intravenous thrombolysis (IVT) is an established treatment in eligible patients which aims to lyse the causal clot and recanalise the occluded vessel, thus re-establishing perfusion of the affected area of brain [2].

Alteplase is a recombinant tissue plasminogen activator (rt-PA) which has been the drug of choice for intravenous thrombolysis since the first positive trials of IVT in the 1990s [3]. Other intravenous thrombolytic agents have been investigated to a limited extent. Streptokinase investigation was abandoned after it did not improve outcomes and was associated with higher bleeding risk in AIS within 6 h of onset in 2 trials in the early 1990s; desmoteplase was investigated in later time windows (3–9 h after onset) in imaging selected patients, but was not found to be effective in a series of small trials [4]. Alteplase significantly improves the outcome from AIS, but has several drawbacks which may make an alternative thrombolytic agent desirable. These include the method of drug administration which is open to error and delay in an emergency setting anbt d the propensity for thrombolysis-related intracerebral haemorrhage [5].

Tenecteplase is a genetically engineered tissue plasminogen activator which has potential to be an alternative to alteplase [4]. In this review, we examine the pharmacology, uses, and evidence base for alteplase and tenecteplase and discuss which may be the thrombolytic of choice in the future for the treatment of AIS.

## Alteplase

Alteplase is a recombinant form of tissue plasminogen activator, a protease found in endothelial cells which catalyses the conversion of plasminogen to plasmin which in turn breaks down the fibrin components of a thrombus [4]. Alteplase has a very short circulating half-life and therefore is administered as a bolus (10% of dose) followed by infusion of the remaining drug over 1 h. Plasma concentration declines rapidly after the initial bolus administration and delays of greater than 5 min in commencing the infusion mean that plasma concentration increases only slowly and may never achieve target concentration[6]. Delays between the bolus and infusion of alteplase are common in routine clinical use, an average of 9 min being documented in one single centre study [7], potentially compromising alteplase efficacy.

The landmark National Institute of Neurological Disorders and Stroke (NINDS) trial published in 1995 [3] demonstrated significantly greater improvement in disability-free recovery for AIS patients treated with alteplase within 3 h after symptom onset compared to placebo. The absolute benefit for excellent functional outcome was around 12%. This led to regulatory approval for the use of alteplase for AIS in addition to its uses in myocardial infarction and pulmonary embolism. Trials of alteplase in different time windows, more varied populations, and in imaging-selected patients have followed. An individual-level meta-analysis of 9 trials involving 6756 patients concluded that alteplase treatment within 4.5 h, irrespective of age or stroke severity, significantly increased the likelihood of a good functional outcome in patients with AIS [8], with the caveat that benefit declines steeply with increasing delay from stroke onset to treatment.

## Tenecteplase

Tenecteplase is a third-generation thrombolytic produced with recombinant DNA technology as a modified form of alteplase with alterations in the protein structure at 3 sites (modified amino acid sites designated by the letters T, N, and K leading to the drug’s alternative name of TNK). These changes prolong the half-life of tenecteplase and allow greater binding affinity for fibrin than alteplase [4]. Tenecteplase is administered as a single bolus making it an attractive alternative in the management of AIS to alteplase. Preparation of a single bolus is simpler and less time consuming in an emergency setting, particularly as AIS patients may require transfer to another hospital site for access to mechanical thrombectomy, and avoid the bolus-infusion delay issue that compromises therapeutic plasma concentrations of alteplase. Avoidance of ongoing infusions may reduce the need for medical supervision during transfer and improve door to needle time in hospitals that utilise MRI rather than CT-based acute stroke imaging protocols.

Animal studies in a rabbit model of embolic stroke found tenecteplase had a wider dose range and longer therapeutic window than alteplase and were associated with better performance in a post-stroke behavioural rating scale [9].

Tenecteplase superseded alteplase in the management of acute myocardial infarction (AMI) after the ASSENT-2 trial demonstrated its non-inferiority [10]. In trials of AMI, tenecteplase (0.5 mg/kg dose) was found to improve recanalisation significantly prior to percutaneous coronary intervention (PCI); however, this came at a cost of a higher rate of major adverse events including intracerebral haemorrhage [11]. In subsequent trials at earlier timepoints, it was found that rates of intracerebral haemorrhage were highest in those aged > 75, prompting a reduction in the dose of tenecteplase to 0.25 mg/kg in this group [12]. Subsequently, no intracerebral haemorrhages occurred at this dose, whilst efficacy in AMI treatment and mortality was comparable [13]. Trials of tenecteplase 0.25 mg/kg in AMI prior to PCI are ongoing [14].

## Tenecteplase vs alteplase in AIS: published studies

The first randomised, controlled trial of tenecteplase in AIS compared alteplase with tenecteplase at 3 different doses, 0.1, 0.25, and 0.4 mg/kg [15]. The study was halted early due to funding and recruitment issues. The trial used an adaptive design with combined safety and early efficacy assessments guiding recruitment at each dose level, and recruitment to the 0.4 mg/kg tenecteplase dose was discontinued after only 19 patients. The low numbers recruited meant no firm conclusions were possible.

Two further phase II trials comparing alteplase and tenecteplase followed. An Australian study compared standard dose alteplase with tenecteplase at either 0.1 or 0.25 mg/kg in patients with AIS presenting within 6 h of symptom onset and specific imaging criteria (vessel occlusion on CT angiography and an ischaemic lesion ≥ 20% greater than core lesion on CT perfusion) [16]. In 75 patients, they found the 0.25 mg/kg was superior to lower dose tenecteplase and to alteplase in reperfusion of the ischaemic lesion, clinical improvement at 24 h, and excellent recovery at 90 days. The ATTEST study compared alteplase with tenecteplase at 0.25 mg/kg in patients eligible for IVT presenting within 4.5 h of symptom onset [17]. In 104 patients, no differences were found between treatments for proportion of penumbra salvaged and any other imaging or clinical outcome.

Individual patient data pooled analysis of these 3 trials found no significant differences between alteplase and tenecteplase at all doses investigated, but suggested that the 0.25 mg/kg dose warranted further investigation as it had the greatest odds of achieving early neurological improvement, excellent functional outcome and avoiding intracerebral haemorrhage compared with alteplase [18]. Subgroup analyses suggested potentially superior efficacy among patients with large vessel occlusion (occlusion of the intracranial internal carotid artery or first part of the middle cerebral artery) [19, 20].

The NOR-TEST trial compared alteplase with tenecteplase 0.4 mg/kg in patients eligible for IVT presenting within 4.5 h of symptoms; or those presenting within 4.5 h of awakening with stroke symptoms and meeting magnetic resonance imaging (MRI) criteria for eligibility; or those eligible for IVT as a bridging therapy to mechanical thrombectomy. Although 1100 patients were recruited, there was a large proportion of stroke mimics (17%), and the majority of patients had mild stroke (median National Institutes of Health Stroke Scale (NIHSS) score of 4). No difference was found between groups in clinical or safety outcomes. In recognition of the confounding issues of mild stroke, the subsequent NOR-TEST-2A trial [21•] was undertaken in patients with AIS of minimum severity (defined as NIHSS score > 5). The trial was discontinued at interim safety review after a four-fold excess of symptomatic intracerebral haemorrhage was observed in the tenecteplase arm (16% tenecteplase versus 4% alteplase), although there were several imbalances in prognostic markers between groups—with tenecteplase arm participants being older (median age 73.2 vs 68.6 years), less likely to have a stroke mimic diagnosis (3% vs 11.5%), and lower levels of baseline functional impairment (40% had modified Rankin Scale (mRS) score ≥ 1 vs 26.9%). Plans for further investigation of the 0.4 mg/kg dose have been abandoned in favour of the 0.25 mg/kg dose.

The EXTEND IA-TNK trial enrolled patients eligible for mechanical thrombectomy within 4.5 h of symptom onset and randomised to alteplase or tenecteplase 0.25 mg/kg [22]. In 202 patients, the primary outcome of reperfusion of ≥ 50% of the vascular territory or absence of the initial occluding thrombus occurred in significantly more patients in the tenecteplase (22%) than the alteplase (10%) group (adjusted odds ratio 2.6 (1.1–5.9) *p* = 0.02). There were no significant differences in patients achieving independent recovery (defined as mRS 0–2 at 90 days) or in early neurological improvement between groups. A follow-up study, EXTEND IA TNK part 2 [23], compared tenecteplase 0.25 and 0.4 mg/kg in a similar study design. No significant differences were found in the radiological primary outcome or other clinical or safety outcomes, inferring that the 0.4 mg/kg offered no advantage over the lower 0.25 mg/kg dose in patients eligible for mechanical thrombectomy.

Superior reperfusion with tenecteplase 0.25 mg/kg compared with alteplase was reported among patients treated in a mobile stroke unit setting in the TASTE-A trial [24] in Melbourne, Australia.

## Tenecteplase: a non-inferior thrombolytic?

In a 2019 meta-analysis of the 5 published alteplase versus tenecteplase trials to that date, Burgos and Saver concluded that non-inferiority had been demonstrated [25•]. They calculated the proportions achieving disability-free survival after AIS of 57.9% with tenecteplase and 55.4% with alteplase with a risk difference of 4% (− 1–8) on meta-analysis. A pre-specified non-inferiority margin of 6.5% as well as secondary margins of 5% and 1.3% were met. However, weaknesses of the meta-analysis included a heterogenous population in terms of stroke severity, a high number of stroke mimics (from the NOR-TEST data), differing doses of tenecteplase, and results largely being driven by trials selecting patients with large vessel occlusion. The primary non-inferiority margin selection was criticised for being drawn from a trial comparing two doses of alteplase [2].

Encouraged by trial and meta-analysis results to date, some stroke centres have elected to implement routine use of tenecteplase. Zhong et al. reported routine use of tenecteplase compared with alteplase at 3 stroke centres in New Zealand as being feasible with similar clinical and safety outcomes [26]. Warach et al. reported plans for a prospective study of use of tenecteplase for all IVT eligible patients presenting within 4.5 h of symptom onset in a 9 hospital network in Texas [27]. In France, the Tenecteplase Treatment in Ischemic Stroke (TETRIS) study group [28] also reported safety, efficacy, and recanalisation rates using tenecteplase routinely to be in line with published results. The move to adopt tenecteplase as routine thrombolytic agent of choice has been spurred on by the COVID-19 pandemic which placed unprecedented pressure on emergency departments globally, motivating calls for use of tenecteplase over alteplase as an easier and more quickly administered alternative [29]. However, this in turn led to shortages of tenecteplase for the management of acute myocardial infarction due to supply issues of the drug in some countries [30].

The alteplase compared to tenecteplase (ACT) trial, the first large trial comparing tenecteplase 0.25 mg with alteplase, was presented in May 2022 [31•]. In 1600 patients randomised within 4.5 h of AIS onset, tenecteplase exhibited an increase in excellent recovery of 2.1% compared to alteplase, meeting the pre-specified non-inferiority margin. The trial found no significant differences in any outcome measure. A subgroup analysis in patients with large vessel occlusion, a trend towards superiority of tenecteplase was seen.

## Current guidelines and ongoing clinical trials of tenecteplase in AIS

A number of clinical trials of tenecteplase in AIS are ongoing covering a variety of IVT indications and timeframes. Current guidelines for different clinical scenarios and future potential applications of tenecteplase currently being investigated in these scenarios are summarised below.

### Disabling stroke < 4.5 h

The European Stroke Organisation guidelines for IVT published in 2021 maintained a recommendation of alteplase over tenecteplase for patients routinely eligible for IVT, but recommended tenecteplase 0.25 mg/kg over alteplase for patients receiving bridging IVT prior to mechanical thrombectomy [2]. The American Heart Association/American Stroke Association guidelines published in 2019 similarly recommended tenecteplase as a suitable alternative to alteplase in the pre-mechanical thrombectomy population [32]. Guidelines from both ESO and AHA acknowledged the low quality of the evidence available at the time of writing, and recommendations were weak. The addition of the ACT trial is likely to strengthen the recommendation of tenecteplase 0.25 g/kg as an alternative to alteplase.

Trials comparing tenecteplase and alteplase in this patient population are ongoing. The ATTEST-2 trial (NCT02814409) is a randomised controlled trial comparing tenecteplase 0.25 mg/kg and alteplase for patients routinely eligible for IVT presenting within 4.5 h of symptom onset. The TASTE trial (ACTRN12613000243718) is comparing tenecteplase 0.25 mg/kg and alteplase for patients with AIS presenting within 4.5 h of symptom onset who have favourable baseline imaging characteristics (confirmed CT perfusion mismatch with ischaemic core < 70 ml).

### Disabling stroke with onset beyond 4.5 h

Patients with AIS may be eligible for thrombolysis beyond the 4.5-h window on the basis of brain imaging. CT angiogram can identify patients with large vessel occlusion, and CT perfusion can identify patients with small ischaemic core (irreversibly damaged, severely hypoperfused tissue) and larger volumes of penumbra (hypoperfused but potentially rescuable tissue), a pattern referred to as “target mismatch”. The EXTEND trial, and a subsequent meta-analysis of the EXTEND, EPITHET, and ECASS-4 trials, demonstrated the efficacy of alteplase in patients with target mismatch on CT perfusion imaging up to 9 h after symptom onset for improving functional outcome after AIS [33, 34]. The current European Stroke Organisation guidelines [2] support the use of alteplase in patients with target mismatch up to 9 h after symptom onset in whom mechanical thrombectomy is not planned.

The TIMELESS trial (NCT03785678) is comparing tenecteplase 0.25 mg/kg and alteplase for patients with AIS presenting between 4.5 and 24 h of symptom onset in whom imaging confirms an anterior circulation stroke (ICA, M1, or M2 occlusion) with favourable perfusion imaging (mismatch with ischaemic core < 70 ml). The ETERNAL trial (NCT04454788) in Australia will also compare tenecteplase and alteplase in patients with LVO and target mismatch.

### Wake-up stroke

Hyperacute MRI can identify patients with potentially recent onset of ischaemia based on the presence of a lesion on diffusion-weighted imaging (DWI) that is not yet abnormal on a T2 fluid-attenuated inversion recovery (FLAIR) sequence (DWI-FLAIR-mismatch) [35]. The WAKE-UP trial demonstrated the efficacy of alteplase in patients with wake-up strokes with DWI-FLAIR mismatch on acute MRI [36]. The EXTEND trial also recruited patients with target mismatch on CT perfusion if they presented with symptoms on wakening up to 9 h from the midpoint of sleep. Accordingly, European guidelines support the use of alteplase in patients fulfilling these imaging criteria if thrombectomy is not planned [2].

The TWIST trial [37] compared tenecteplase 0.25 mg/kg against non-IVT standard of care for patients with wake-up stroke presenting within 4.5 h of wakening, enrolled on the basis of a non-contrast CT compatible with IVT. The trial stopped short of its planned sample size and was unable to establish non-inferiority or superiority of tenecteplase for any outcome measure.

### Minor stroke and TIA

There is currently no standard definition of “minor stroke”, and total scores on clinical assessments scales such as the NIHSS do not reliably distinguish disabling from non-disabling deficits. Trials of alteplase generally required the presence of a disabling neurological deficit and excluded patients with non-disabling or rapidly improving deficits, but these terms were open to individual interpretation. European and American guidelines recommend treatment of minor stroke with disabling symptoms with alteplase [2, 32] as this group accounted for around 10% of patients in the alteplase trials [8]. The American guidelines suggested tenecteplase 0.4 mg/kg as a potential alternative to alteplase in patients with minor neurological impairment and no major intracranial occlusion [32], based on the NOR-TEST findings, with a weak recommendation, but this is likely to be superseded by NOR-TEST-2A results. However, IVT is not recommended with minor symptoms which are non-disabling by either guideline, since such patients were excluded from the main alteplase trials. The PRISMS trial randomised patients with non-disabling symptoms and NIHSS 0–5 to aspirin or alteplase within 3 h of symptom onset and found no benefit of alteplase, although the trial was terminated early with only around one-third of the planned sample size [38]. Presently, guidelines recommend dual antiplatelet therapy with aspirin and clopidogrel in this patient group [39, 40].

The TEMPO-1 study was a dose escalation study of tenecteplase in patients with minor stroke or TIA (NIHSS 0–5, non-disabling symptoms) and confirmed LVO [41]. Tenecteplase was found to be safe at 0.1 and 0.25 mg/kg doses. Complete recanalisation was strongly predictive of subsequent excellent functional outcome. The TEMPO-2 trial (NCT02398656) is comparing tenecteplase 0.25 mg/kg against standard of care in patients presenting with minor stroke or TIA (NIHSS 0–5) who have a confirmed LVO.

## Conclusions

Tenecteplase has pharmacological properties that suggest potential advantages over alteplase. The dose of 0.25 mg/kg (to a maximum of 25 mg) has evidence of improved reperfusion compared to alteplase in selected patients with large vessel occlusion. The improved early reperfusion in the EXTEND-IA TNK trial was accompanied by better clinical outcomes, leading to recommendations to consider tenecteplase as the thrombolytic of choice in patients with large vessel occlusion. Since it is desirable to initiate thrombolysis as rapidly as possible, delaying thrombolytic administration until after confirmation of large vessel occlusion by CT angiography is not ideal, however, and challenging for optimal clinical workflow. There would be clear benefit from a single thrombolytic agent being recommended for all AIS patients.

Considerable enthusiasm exists for use of tenecteplase in AIS to replace alteplase as the thrombolytic agent for routine practice, driven primarily by the practical benefits over alteplase in ease of administration by single bolus that are especially advantageous in the common scenario of inter-hospital transfer. The published evidence base prior to 2022 among unselected thrombolysis-eligible patients with AIS consisted of three small phase II trials and one phase III trial (NOR-TEST), with the complication that NOR-TEST used the 0.4 mg/kg dose subsequently abandoned on safety grounds and recruited a large proportion of minor strokes and stroke mimics. Consequently, guidelines did not recommend tenecteplase over alteplase for general use. The recent data from the ACT trial showing non-inferiority of the 0.25 mg/kg dose is likely to strengthen recommendations towards tenecteplase, but further ongoing trials in the relevant population will report in the next 12–18 months. Even if guideline recommendations move in favour of tenecteplase for thrombolysis in all AIS patients, regulatory approval for tenecteplase would be advantageous compared to widespread off-label use [42]. Tenecteplase is currently packaged for the higher dose used in acute myocardial infarction management, including weight-graduated syringes and dose instructions, leading to the potential for dosing errors in the AIS population [43]. In addition, a large amount of drug is inevitably wasted when stroke doses—maximum of half of a 50-mg vial—are prepared. Manufacture of a stroke-specific dose with appropriate packaging, and secure drug supply sufficient for widespread AIS use, may depend on regulatory approval of tenecteplase for the AIS indication.

Further extension of tenecteplase indications in stroke may follow in future based on the multiple ongoing clinical trials in situations including extended time windows, minor stroke, and TIA.

## Competing Interests

Participation in advisory boards for Boehringer Ingelheim; lecture fees from Boehringer Ingelheim; and consultancy fees from Boehringer Ingelheim.
